# ETHNICITY AND WELL-BEING AMONG OLDER IMMIGRANTS IN DIVERSE SOCIOCULTURAL CONTEXTS

**DOI:** 10.1093/geroni/igae098.0624

**Published:** 2024-12-31

**Authors:** Daniel W L Lai

**Affiliations:** Chair

## Abstract

The critical interaction between culture, ethnicity, and social determinants of health is a vital area of study, particularly regarding the unique experiences of ethnocultural minority older adults. The complex intersections of these factors across diverse social landscapes are ripe for investigation to uncover inequalities and inform targeted strategies to reduce the inequalities encountered by these groups. This symposium presents four key studies that examine the well-being, healthcare access, and civic participation of aging Asian immigrants, with a focus on South Asian populations in Hong Kong and Canada, as well as Chinese communities in Canada and the United States. These studies highlight the need for culturally congruent interventions. They emphasise the challenges faced in health and mental health service accessibility and the rich tapestry of cultural diversity within civic involvement. Critical factors such as the quality of intergenerational relationships, the breadth and depth of social capital, language skills, transportation availability, financial dependencies, and the impact of pre-migration histories are shown to significantly affect the lives and civic engagement of aging Asian immigrants. The research presented here strongly advocates for culturally attuned policies and practices. It underscores an imperative for health promotion, healthcare accessibility, and civic inclusion that are responsive to the cultural identities and preferences of diverse older populations. The findings of these studies are critical to the development of targeted interventions, public policies and supportive frameworks that address the unique needs and overcome the barriers faced by ageing migrants around the world.

